# The positive effect of moral self-concept on fraudulent behavior and the need for moral cleansing

**DOI:** 10.1038/s41598-025-16403-9

**Published:** 2025-08-29

**Authors:** Tamás Keller, Péter Szakál

**Affiliations:** 1 ELTE Centre for Social Sciences, Computational Social Science Research Group, Tóth Kálmán utca 4., Budapest, 1097 Hungary; 2 ELTE Centre for Economic and Regional Studies, Institute of Economics, Tóth Kálmán utca 4., Budapest, 1097 Hungary; 3 University of Szeged, Szeged, Hungary

**Keywords:** Human behaviour, Computational science

## Abstract

Both moral cleansing and moral licensing theories suggest that moral self-concept positively influences subsequent engagement in fraudulent behavior. Specifically, a decrease in moral self-concept reduces dishonesty (moral cleansing), while an increase promotes it (moral licensing). However, within these theoretical frameworks, prior research has rarely measured moral self-concept directly, and even fewer studies have manipulated it experimentally. As a result, a direct test of the role of self-concept in these theories is still lacking. This study addresses this gap by experimentally manipulating and directly measuring moral self-concept to examine its relation to subsequent fraudulent behavior. A large-scale randomized experiment was conducted among Hungarian university students, using a subtle priming intervention that urged participants to recall their past moral transgressions. This manipulation effectively lowered moral self-concept, which in turn reduced dishonest behavior, as measured by the fraudulent misreporting of numbers rolled in a monetarily incentivized “die-under-the-cup” task. This causal chain aligns with the theory of moral cleansing. Using the randomized treatment as an instrument, the study has identified a positive causal effect of moral self-concept on fraudulent behavior, indicating that a decrease in moral self-concept reduces fraudulent behavior.

**Supplementary Information:**

The online version contains supplementary material available at 10.1038/s41598-025-16403-9.

## Introduction

*Hubris*—an arrogant and stubborn pride rooted in an overly positive self-view without humility—was considered the greatest sin against the gods in ancient Greek mythology, often leading mythological characters to their downfall as they engaged in unethical behavior. Ever since ancient times, exploring how self-perception shapes behavior has remained a key focus of research^1–5^. Specifically, as people infer their moral character by reflecting on past actions^6^, a key question in the recent economics and psychology literature is how moral self-concept—perception of one’s own morality—shapes future fraudulent behavior^7–9^.

Four possible answers to this question have been outlined in previous scholarship inasmuch as past actions can either strengthen or weaken one’s moral self-concept—ethical behavior reinforces it, while unethical behavior does the opposite—and both an increase and a decrease in moral self-concept can either encourage or discourage future fraudulent behavior.

On the one hand, having engaged in ethical behavior in the past can improve self-concept. This may lead individuals to resist temptation and act honestly in the future, resulting in less dishonesty—an effect known as positive moral reinforcement^10^. However, an enhanced moral self-concept may also create a sense of entitlement, leading to greater dishonesty through moral licensing, as individuals perceive a surplus of moral credentials that may make them feel entitled to commit deception^11,12^.

On the other hand, past unethical behavior can lower moral self-concept. A decrease in self-concept may prompt corrective actions aimed at cleansing oneself of past wrongdoings, a process known as moral cleansing^13,14^, which has the effect of reduced dishonesty later on. However, a decreased moral self-concept may also reinforce further unethical behavior, as individuals who view themselves as morally compromised seek consistency through negative moral reinforcement, thus leading to greater dishonesty^15^.

Moral balance theory^16^ has situated moral licensing and moral cleansing in a dynamic framework. It is argued that before people commit fraudulent behavior, they calculate a balance from their past morally relevant actions and compare this balance to their fundamental conception of themselves (labeled as the level of acceptable morality), from which they do not wish to depart. If this balance is positive, they can allow themselves to participate in fraudulent behavior since they have a surplus of positive self-concepts (moral licensing). However, if their moral balance is negative, they cannot allow themselves to engage in fraudulent behavior since they have a deficit of positive self-concepts, which might be further diminished by committing fraudulent behavior once again. Therefore, they refrain from fraudulent behavior and instead choose to act ethically (moral cleansing).

Extending the logic of moral balance theory, one can hypothesize a positive relationship between self-concept and fraudulent behavior. Specifically, a decrease in moral self-concept leads to reduced fraudulent behavior (moral cleansing), while an increase in self-concept leads to increased fraudulent behavior (moral licensing). This may also suggest that the more individuals perceive themselves as honest, the more likely they are to engage in unethical behavior, whereas those who view themselves as less honest may be more inclined to act ethically.

This study focuses on the effects of a decreased moral self-concept, which, according to theory, can either reduce fraudulent behavior (as suggested by moral cleansing) or increase it (through negative moral reinforcement). Beyond distinguishing between these two possibilities, the primary aim of the study is to identify the *causal* effect of moral self-concept on fraudulent behavior—a topic that has received relatively little attention in prior research. While the theory of moral cleansing has been explored extensively, studies within this framework have typically not measured moral self-concept directly. For instance, in one study, White participants who were falsely informed that they held racial biases donated more to Black panhandlers than to White ones^17^. Similarly, drivers who received false feedback that indicated they had engaged in reckless driving expressed greater interest in community service^18^. In another study, participants who wrote self-relevant stories using negative traits donated more to charity than those who described themselves using positive qualities^19^. In all these cases, the role of moral self-concept was inferred rather than explicitly measured.

Generally, only a few empirical studies have directly assessed moral self-concept, and even fewer have manipulated it experimentally to establish a causal relationship. Therefore, the assumption that a decrease in moral self-concept results in a decrease in subsequent fraudulent behavior has rarely been tested in prior research. Instead, most prior research has inferred moral self-concept from prior behavior^10,20^ or referenced it indirectly—for example, by comparing the effects of writing about one’s own positive or negative traits, which is thought to cause an increase or a decrease self-concept, to writing about someone else’s traits, which presumably exerts neither of these effects^19^. Other studies have examined constructs related to self-concept, such as the centrality or accessibility of moral identity^2,8^—reflecting the importance of morality and the extent to which people see themselves as moral among their many possible identities—as well as loosely related concepts like feelings of authenticity^15^ and current emotional state^21^. Even the seminal paper that introduced the concept of moral balancing^16^ merely inferred moral self-concept from past behavior rather than measuring it directly. As a result, little is known about how exogenously manipulated perceptions of people’s own morality affect their fraudulent behavior.

Therefore, this study combines two steps. First, it specifically tests whether subtle, randomized reminders of one’s own past moral transgressions decrease moral self-concept. Then it asks whether a reduced moral self-concept decreases the likelihood of engaging in fraudulent behavior.

A large-scale, randomized experiment was conducted among university students at the University of Szeged, Hungary’s second-largest university, to investigate how exogenously decreased moral self-concept affects engagement in subsequent fraudulent behavior. The 2,194 students involved were randomly assigned to treatment and control conditions. In the treatment group, students’ moral self-concept was experimentally manipulated through priming questions about common fraudulent behaviors where people typically fail to act ethically, which prompted participants to recall their own past moral transgressions. The treatment questions were followed by a direct assessment of moral self-concept, deploying the simple question: “How honest do you think you are?” The self-concept question was followed by a modified version of the “die-under-the-cup” task^22^, in which students were incentivized to behave fraudulently by overreporting the number they had rolled, thereby increasing their chances of winning a gift voucher worth approximately $10 in local currency.

Overall, in the treatment group, the sequence of questions was as follows: treatment questions, a self-concept assessment, and a measurement of fraudulent behavior. The control group followed the same procedure but without the treatment questions. Consequently, control students were not primed and reminded of their past moral transgressions at the start of the experiment. As reminders of past transgressions are thought to diminish moral self-concept by evoking feelings of personal contamination^13,14,23^, the treatment was designed to lower students’ moral self-concept exogenously, allowing us to examine how this reduction influences subsequent engagement in fraudulent behavior.

The results show that students randomized to the treatment group have a lower moral self-concept than those in the control group, as treated students rated themselves as less honest on the self-concept question. This exogenous decrease in moral self-concept, in turn, led to less deception in the die-under-the-cup task. Taken together, these findings align with predictions of the moral cleansing theory. Furthermore, using the randomized treatment assignment as an instrument for self-concept^24^, the instrumental variable estimation confirms that the decline in fraudulent behavior was specifically driven by the decrease in moral self-concept. Thus, the study establishes a positive relationship between moral self-concept and fraudulent behavior, as predicted by both moral cleansing and moral licensing theories.

In sum, there is a downside to an overly positive self-concept: it fuels dishonesty. In contrast, recalling past moral transgressions fosters humility and curbs unethical behavior.

## Materials

The study has been reviewed and approved by the Institutional Review Board (IRB) at the ELTE Centre for Social Sciences, Budapest. All research was conducted in accordance with the relevant guidelines and regulations. Informed consent was obtained from all participants before data collection. No underage students took part in this study.

We conducted a large-scale randomized experiment at the University of Szeged between May 6 and October 6, 2021. An invitation e-mail was sent to all active university students to recruit participants, followed by reminder e-mails throughout the study period. Our initial recruitment e-mail reached 17,408 students, with 3,668 responding. Of these, 2,203 students participated, resulting in a university-wide participation rate of 12.7% (2,203/17,408). The total sample in the recent experiment consists of 2,194 students, as nine were excluded for providing invalid responses in the “die-under-the-cup” task, such as reporting numbers outside the feasible range of 1 to 6.

The experiment was embedded in a computer-based online survey and consisted of the following sequential blocks: treatment questions, a self-concept measurement, a die-under-the-cup task, and additional questions about students’ social and health backgrounds. On average, it took approximately 20 min to complete.

The treatment involved a brief, four-item questionnaire designed to remind students of their past moral transgressions. It listed common, everyday forms of dishonest behavior (e.g., cheating for better grades), and students were asked to place their own actions on a scale ranging from unethical to ethical. The purpose of the treatment was to subtly prompt reflection on past unethical behavior. Since most—if not all—students are likely to have engaged in some of the behaviors listed, this priming was expected to subconsciously evoke memories of past moral transgressions, thereby lowering moral self-concept^13,14,21^.

The treatment was randomly allocated, with 80% of the students assigned to the treatment group and 20% to the control group. The students in the treated and control groups are well balanced in terms of baseline variables (see Table A1 in the Appendix). The differences between the treated and control groups are substantively small and statistically not significant.

Moral self-concept was assessed using a single question: “How honest do you think you are?” Students responded using a scale from 0 to 10, where 0 means “I’m not honest at all” and 10 means “I’m extremely honest.” The treated students answered this question after the treatment, while this was the first question in the experiment for the control students.

Fraudulent behavior was measured with a modified version of the incentivized die-under-the-cup task^22^. In the standard task, participants secretly roll a six-sided die and report the outcome, with higher reported numbers leading to higher monetary rewards. Because rolls are unobserved, participants can misreport their numbers without the risk of being caught. While individual cheating cannot be directly detected, aggregate cheating can be inferred by comparing the distribution of reported numbers to the expected theoretical distribution.

We modified the standard die-under-the-cup task by asking participants to roll a virtual die ten times in succession. This allowed us to assess individual deceptive behavior by comparing the distribution of reported outcomes to the theoretical distribution of ten independent die rolls. Students were monetarily incentivized to report higher numbers than they actually rolled, as their chances of winning a gift voucher corresponded to the total of the reported number (not the rolled number) in the die-under-the-cup task. Thus, the more the reported sums deviated upward from the expected value of ten independent die rolls, the more likely individual students committed deception, as higher sums occur with lower probability. Our primary measure of fraudulent behavior was the sum of the ten rolls reported, ranging from 10 (10 × 1) to 60 (10 × 6).

Table 1 presents descriptive statistics for the key variables in the analysis.

**Table 1:** Descriptive statistics: mean and standard deviation (SD) for the main variables.

	Fraudulent behavior: The sum of the ten rolls (range: 10–60)	Moral self-concept (range: 0–10)	Share of female students	Age (in years)
The full sample				
Mean	36.53	7.95	0.73	23.95
SD	6.35	1.38	0.44	6.54
N	2,194	2,194	2,194	2,194
Treated students				
Mean	36.38	7.84	0.74	24.06
SD	6.29	1.40	0.44	6.60
N	1,767	1,767	1,767	1,767
Control students				
Mean	37.14	8.40	0.70	23.52
SD	6.54	1.19	0.46	6.28
N	427	427	427	427

## Results

### The causal treatment effect on moral self-concept

The average student exhibited a relatively high moral self-concept, with a mean score of 7.9 on a scale of 0–10 (SD = 1.4), reflecting a right-skewed distribution. Very few students considered themselves highly dishonest, with only one student reporting a score of 0 and two students reporting a score of 1.

**Fig. 1:**
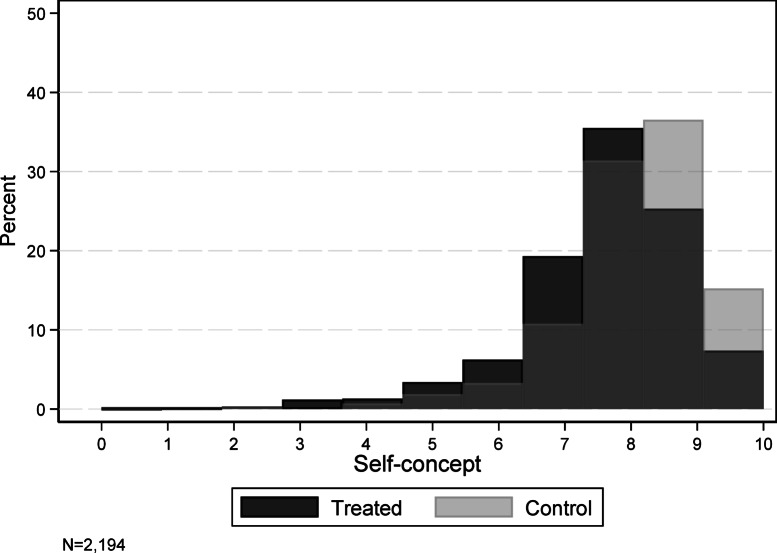
Distribution of self-reported moral self-concept in the treatment and control groups by response frequency.

As expected, the treatment reduced students’ moral self-concept. The treated students reported lower moral self-concept compared to the control group. Figure 1 illustrates this effect: the highest possible rating of self-concept (10) was nearly twice as common among control students (light gray) as in the treatment group (dark gray). Similarly, the second-highest rating of self-concept (9) was reported 11% points more frequently in the control group. In contrast, all lower ratings appeared more frequently among treated students, indicating a systematic downward shift in self-concept due to the treatment.

Column 1 in Table 2 presents multivariate regression results with regard to the causal effect of the treatment on students’ moral self-concept. Treated students reported a lower moral self-concept than control students by 0.5 units, corresponding to a − 0.36 effect in standard deviation (SD) units, which is a medium-sized effect (*p* < 0.001). The treatment effect is notable compared to other control variables. Except for gender, none of the control variables significantly influenced self-concept. Female students reported 0.21 units higher self-concept than male students, which accounts for approximately 40% of the treatment effect on self-concept (*p* = 0.009).

## The causal treatment effect on fraudulent behavior

**Fig. 2:**
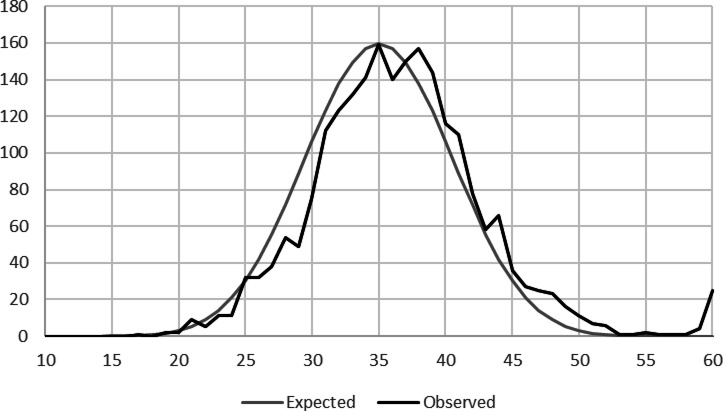
The number of students (y-axis) who reported (black line) a given number rolled as the sum of the ten die rolls (x-axis) and the theoretical statistical distribution of the sum of the ten die rolls (gray line).

Figure 2 presents the distribution of the outcome variable: the sum of the ten die rolls reported by students. The gray line represents the expected distribution based on ten independent rolls. The observed distribution (black line) is slightly right-shifted relative to the theoretical distribution, indicating that students were more likely to report higher numbers than lower numbers. This pattern suggests that incentivization worked as intended. As higher reported numbers increased students’ chances of winning, they were motivated to overreport the numbers rolled, whereas there was no motivation to underreport. In line with this reasoning, the black line falls consistently below the gray line for values below 35 on the x-axis.

Overall, students only cheated to a limited extent, consistent with findings in experimental economics, psychology, and sociology, suggesting that people typically cheat far less than they could^25^. For instance, while the expected mean sum of ten independent die rolls is 35, the mean observed in our sample is 36.5 (SD = 6.4). Notably, from a sum of 38 onward, there are 202 more students than expected, indicating that at least 9% (202/2,194) of students overreported their rolls. A particularly striking feature of the distribution is the peak at the highest possible value of 60. Given the sample size of 2,194 students, the expected number of students reporting this outcome should be nearly zero. However, 25 students claimed to have rolled the maximum sum.

Importantly, the mean sum of the ten die rolls is 37.14 in the control group, while the mean for the treatment group is 0.76 units less at 36.38. The bivariate difference between the mean for the treated and control groups is statistically significant (*p* = 0.03). Despite the reduction caused by the treatment, the mean in the treatment group still exceeds what would be expected from a normal distribution of ten independent rolls (the difference between 36.38 and 35 is statistically significant, *p* < 0.01), indicating that treated students still engaged in some fraudulent behavior, though at a lower level than control students did. Thus, the treatment reduced fraudulent behavior but did not eliminate it.

Column 2 in Table 2 shows the causal effect of treatment on fraudulent behavior using multivariate regression analysis. Treated students reported rolling smaller numbers than control students. The difference in the sum of the ten die rolls between the treated and control groups is 0.78—a small nominal effect that is statistically significant at the 5% level (*p* = 0.046). Expressed in SD units, this also corresponds to a small effect size of − 0.12. However, the treatment effect accounts for 36% (0.78/2.14) of the difference between the expected value of ten independent die rolls (35) and the control group mean (37.14), demonstrating a sizable reduction in fraudulent behavior in the treatment group compared to the control group. Taken together, Columns 1 and 2 indicate that the treatment first reduced students’ moral self-concept, followed by a drop in fraudulent behavior—a finding that aligns with moral cleansing^13,14^.

## The causal effect of moral self-concept on fraudulent behavior

Column 3 in Table 2 shifts the analysis from estimating the treatment effect to addressing the more theoretical question of the causal relationship between self-concept and fraudulent behavior. Since moral self-concept was not randomized and is therefore endogenous, the randomized treatment serves as an instrument for self-concept in the instrumental variable (IV) estimation^24^. That is, the IV analysis focuses on the part of self-concept that responded to the randomized treatment, examining how the exogenously manipulated self-concept influences fraudulent behavior.

Results from the IV estimation show that a one-unit increase in the 11th -grade self-concept measure increased fraudulent behavior by 1.55 units (*p* = 0.041). Consequently, one standard deviation increase in self-reported honesty (SD = 1.39) led to an increase of 2.15 units (1.55 × 1.39) in the sum of reported dice rolls. This represents a small nominal increase within the range of the dependent variable (10 to 60), corresponding to a mid-sized effect of 0.24 SD units.

**Table 2:** Regression results: the causal effect of treatment on self-concept (Column 1) and the (causal) effect of self-concept on fraudulent behavior OLS (Column 2) and IV (Column 3) regressions.

	(1)	(2)	(3)
Dependent variable	Self-concept	Fraudulent behavior	Fraudulent behavior
Model	OLS regression	OLS regression	Instrumental variable regression
Treated	–0.50**	–0.78*	
	(0.08)	(0.39)	
	[–0.36]	[–0.12]	
Self-concept			1.55*
			(0.76)
			[0.24]
Constant	8.04**	39.75**	26.80**
	(0.26)	(1.23)	(8.46)
Observations	2,194	2,194	2,194
R-squared	0.19	0.14	0.02

Standard errors in parentheses, ***p < 0.005, **p < 0.01, *p < 0.05.

Cohen’s d effect sizes (the coefficient divided by the pooled standard deviation of the dependent variable) are in square brackets.

Control variables included in all the models: gender, age, GPA, type/financing/level of educational program, and study program fixed effects. See descriptive statistics for control variables in Appendix A.

## Discussion

We have reported the results of an experiment in which university students’ moral self-concept was manipulated exogenously, inducing a decrease in it. This reduction in self-concept subsequently led to a decrease in fraudulent behavior, supporting the idea of moral cleansing^13,14,21^. The instrumental variable analysis showed that the decrease in moral self-concept causally reduced fraudulent behavior, establishing a positive relationship between the two, consistent with the predictions of both moral cleansing and moral licensing. While the treatment effects on both self-concept and fraudulent behavior, as well as the causal effect of moral self-concept on fraudulent behavior, were all modest, they are meaningful in context: the manipulation was subtle, and the sample exhibited limited variation in fraudulent behavior. Under these conditions, even small effects have substantial significance.

The novelty of this study lies in experimentally manipulating and directly measuring moral self-concept, thereby establishing a causal relationship between self-concept and fraudulent behavior. This represents an advancement compared to prior research that has mostly inferred self-concept rather than measuring it directly^10,19,20^, leaving the causal relationship between self-concept and fraudulent behavior assumed but untested within the specific theoretical frameworks^15,23,26^.

The results of this study clarify and extend previous research on how moral self-concept influences future fraudulent behavior. First, the findings show that a decrease in moral self-concept reduces subsequent dishonesty, rather than increasing it. This supports the moral cleansing perspective^14^ rather than the negative moral reinforcement mechanism, suggesting that feeling morally compromised leads individuals to maintain consistency by engaging in further unethical behavior^15^.

Second, the study tests a shared prediction of both moral cleansing and moral licensing theories—integrated within the broader framework of moral balancing—that moral self-concept positively influences fraudulent behavior. In this regard, the study provides causal evidence to support this positive relationship.

Third, unlike previous research that has focused primarily on moral judgments^16,23^ or intention to donate to charity^19^, the present study represents an advancement over prior literature, as the outcome measured here is actual behavior rather than attitude—and a different type of behavior than previously studied, which mainly focused on prosocial behavior, such as generosity^17,18^. The results presented here, therefore, suggest that decreased moral self-concept not only encourages people to do more good (prosocial) deeds but also motivates them to engage in fewer bad (fraudulent) deeds.

Fourth, this study has analyzed data from over 2,000 students—a substantially larger sample than those of most previous studies, which often involved fewer than 100 participants and^17^, in many cases, even fewer than 50^19,21,23^. The findings thus presented here are likely less vulnerable to small-sample bias, a limitation that may have undermined the credibility of some earlier research^27^.

Fifth, the findings extend prior work by showing that subtle exposure to one’s own unethical behavior—via a brief questionnaire about common moral transgressions—can experimentally lower moral self-concept. This suggests that, beyond recalling past good or bad deeds^21^ or producing self-related texts involving positive or negative traits, reflecting on situations where people commonly fail to act ethically^19^ offers a subtle yet effective method for experimentally manipulating moral self-concept.

Lastly, the results underscore the complexity of how morality influences behavior. While moral priming (e.g., referencing the Ten Commandments) enhances the current accessibility of moral identity within the working self-concept and thereby increases intentions to behave ethically^8^, perceiving oneself as highly moral—i.e., having a heightened moral self-concept—actually decreases ethical behavior and increases fraudulent behavior. This suggests that accessibility of moral identity and self-concept of one’s own morality may exert opposing influences on ethical behavior: the former boosts moral conduct, while the latter may encourage dishonesty.

The results imply that interventions aimed at tempering people’s default positive self-perception could be worth exploring to curb fraudulent behavior. However, the specific design of such interventions requires careful consideration. The intervention we implemented subtly and gently lowered moral self-concept in a way that remained unconscious to the participants themselves. In contrast, direct and intensive interventions that diminish self-concept through humiliation are neither desirable nor advisable and should be avoided.

Reminders represent a commonly deployed approach in the Western cultural heritage to cultivate humility and encourage people to turn away from sin. A widely used reminder is *memento mori*—“remember you will die.” For example, the early Christian author Tertullian describes in his book *Apologeticum* how Roman generals returning from victorious battles were followed by a servant whispering the words: *Respice post te! Hominem te memento!* (“Look behind you! Remember, you are a man!”). At the height of their glory—in a moment when hubris comes naturally—this line served as a reminder that god-like adoration would soon fade, while mortality remained unchanged. Thus, *memento mori*, this simple yet profound phrase, is not meant to instill fear but to encourage humility and reflection by prompting them to confront the limits of eternal life.

A similar strategy is used in Christian liturgy at the start of Lent. On Ash Wednesday, the priest or other cleric draws a cross of ash on a worshipper’s forehead, saying either “Remember that you are dust, and to dust you shall return” or “Turn away from sin and be faithful to the Gospel.” The interchangeability of these two phrases suggests that *memento mori* reminders have long been used to cultivate humility and encourage moral behavior.

In prior experimental research, the most common approach to fostering humility has been to prompt individuals to recall their own past moral transgressions^10,21^. In addition, other effective methods have included reminders of broken promises^13^ or the use of false feedback designed to trigger critical self-reflection^17,18^. Nevertheless, these reminders should focus on people’s own moral transgressions rather than those committed by others. For example, exposure to peers’ unethical behavior^28^ or reading about others’ moral transgressions^21^ could potentially encourage fraudulent behavior rather than reduce it.

The limitations of this study prevent us from drawing a general conclusion about the causal effect of self-concept on fraudulent behavior for two reasons. First, the treatment merely induced a random decrease in self-concept and did not generate a corresponding random increase. As a result, only one of the two necessary conditions for fully examining self-concept manipulation was addressed within the scope of this study.

Second, one should know more about the scope conditions that might influence the link between self-concept and fraudulent behavior. At least three design elements of the current study warrant further examination of potential scope conditions that may limit the generalizability of the findings.

To begin with, in this study, we examined cheating that could not be revealed, since students rolled in secret; thus, there was no fear of being caught. It could be that the connection between self-concept and fraudulent behavior differs if people face a real danger, meaning that their fraudulent behavior can be detected.

Furthermore, in this study, people had low incentives to cheat. In monetary terms, there was little benefit to cheating (≈$10). Moreover, by misreporting the number rolled, students only managed to achieve a slight increase in the likelihood of winning without ensuring a guaranteed reward. The relation between self-concept and fraudulent behavior potentially differs (1) depending on the gain to be achieved by the fraudulent behavior and (2) the contribution of fraudulent behavior to achieving the desired gain.

Lastly, it is possible that our sample, which consists of university students, affected the link explored between self-concept and fraudulent behavior. Therefore, replicating the same experiment among younger people (for example, with students in primary or secondary school) could potentially alter the conclusion that emerged from this study.

Despite these limitations, the study’s findings offer clear insights into the relationship between moral self-concept and ethical behavior. An overly positive self-concept encourages fraudulent behavior, suggesting that hubris fosters unethical behavior. However, reminding individuals of past moral transgressions can serve as corrective leverage, promoting humility and reducing dishonest behavior. In sum, the results presented in this study align closely with the words of the blind prophet Tiresias, who blamed King Creon for his hubris in Sophocles’ *Antigone*:

It’s common knowledge, any human being can go wrong.

But even when he does, a man may still succeed:

He may have his share of luck and good advice.

But only if he’s willing to bend and find a cure.

For the trouble he’s caused. It’s only being stubborn.

Proves you’re a fool^29^.

### Methods

#### The treatment

The treatment consisted of a short, four-item questionnaire that presents two contrasting behavioral extremes: unethical and ethical behavior. The unethical extreme included behaviors such as cheating for better grades, academic plagiarism, pretending to be honest, and only following rules when it is beneficial. Conversely, the ethical extreme encompassed behaviors such as avoiding academic dishonesty, maintaining originality, demonstrating genuine honesty, and adhering to rules even when it is personally disadvantageous. Respondents were asked to position their own behavior on a scale from 1 to 10, where lower values (1–5) were visually associated with the unethical extreme and higher values (6–10) with the ethical extreme. Appendix B provides the translated English version of the questionnaire, while Appendix C summarizes the original Hungarian version.

The purpose of the treatment transcends gathering questionnaire responses; it aims to revive moral awareness that may fade from active awareness and become dormant, thereby losing its influence on behavior^8^. By priming students with ethical dilemmas in which most people fail to act ethically, the treatment indirectly reminds individuals of their past moral transgressions. This recall of past transgressions, even unconsciously, can lead to a decrease in moral self-concept as individuals realize that they have not always behaved ethically.

For example, confronting students with a scenario involving academic cheating might lead them to realize—without consciously admitting it—that they have not always acted ethically, as most people have engaged in some form of cheating in their lives to some extent. Thus, merely completing the questionnaire could unconsciously remind students of their past moral transgressions, leading to a decrease in self-concept^13,14^. Priming people to recognize that they have not always acted morally can lead to a decline in their moral self-concept, which is typically biased in a positive direction, as people tend to overestimate their own virtues^30,31^.

Priming has been widely used in prior research to evoke moral awareness. For example, some experiments have activated moral awareness by having participants unscramble morally charged sentences^32^. Others have directed attention away from money and toward time, arguing that money-priming fosters self-interest, whereas time-priming encourages self-reflection^33^. Another approach primed participants using nouns (“Please don’t be a cheater”) instead of verbs (“Please don’t cheat”), as nouns have stronger implications for identity, while verbs focus more on behavior^26,34^.

#### The measurement of fraudulent behavior

We used the following procedure to measure fraudulent behavior. Students were asked to roll a virtual die ten times in succession. They did this by clicking on web links that opened a virtual die in a new browser window. To ensure complete anonymity, we also encouraged students to open the links on different devices or use their own physical die instead. Providing multiple rolling options reassured participants that their actual rolls could not be tracked.

After each roll, students entered the outcome on the experiment’s webpage. They were informed that each number corresponded to a specific point value: a roll of 1 earned 0 points; 2 earned 1 point; 3 earned 2 points; 4 earned 3 points; 5 earned 4 points; and 6 earned 5 points. The total points accumulated over ten rounds determined their chances of winning a voucher worth Ft3,000 (≈$10) in a lottery, with a maximum winning probability of 50% (10 × 5). Thus, participating students were incentivized to cheat to increase their likelihood of winning the gift voucher.

Throughout the task, we emphasized that students’ chances of winning depended on the numbers they reported. We deliberately used distinct language for numbers “rolled” and “reported” in the instructions when explaining the task to the students. Appendix D provides the translated English version of the experimental measure of fraudulent behavior, while Appendix E summarizes the original Hungarian version.

The numbers rolled remained private: researchers were only able to access the numbers students reported but had no way of verifying the actual rolls. This ensured that fraudulent behavior remained undetectable at the individual level. However, cheating could be inferred at the individual level by comparing the numbers reported to the statistical distribution of ten independent die rolls. Since larger numbers occur less frequently in a fair distribution, an upward deviation from the expected average value suggests dishonest reporting.

#### The instrumental variable approach

**Fig. 3:**
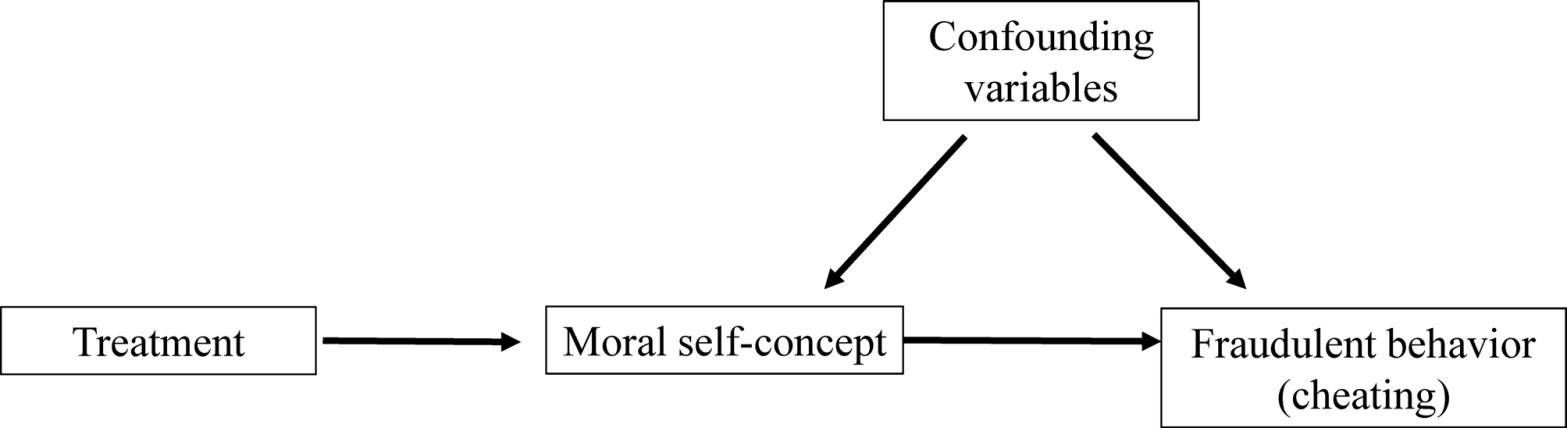
The assumed connection between the key variables in the analysis.

Figure 3 illustrates the concept of the instrumental variable (IV) approach, a method used to examine the causal relationship between self-concept and fraudulent behavior. This approach is necessary because uncontrolled confounders—such as socialization, social norms, peer pressure, and institutional climate—complicate causal inference by simultaneously influencing both self-concept and fraudulent behavior, thus making it difficult to establish a causal relationship between them. To circumvent the concern about uncontrolled confounders, the randomized treatment serves as an instrument for self-concept^24^.

An instrument should meet certain requirements to qualify as such, i.e., relevance and exclusion restriction criteria^35^. First, the randomized treatment is a relevant instrument for self-concept, since the treated students reported lower self-concept (see Fig. 1).

Second, treatment must influence fraudulent behavior solely through its effect on self-concept and not through other channels. While at least two alternative mechanisms could potentially transmit the treatment effect, they can be ruled out in the context of our experiment.

One possible alternative mechanism is that treatment could increase fraudulent behavior through ego depletion^36,37^. Completing the questionnaire requires concentration and self-control, which might leave treated students more vulnerable to temptation in the subsequent die roll exercise. However, the ego depletion mechanism is unlikely to work in this case, as the treatment questionnaire only contained four items. Prior research has shown that ego depletion only has a minor effect on deceptive behavior, even after a much longer (20-minute) questionnaire^38^.

Another possible explanation is that the treatment may have primed participants to behave in a certain way by activating specific mental concepts that encourage either ethical or unethical behavior^39^. However, since our treatment involved rating one’s own behavior on a scale from unethical to ethical, it did not explicitly prime either type of behavior. In particular, prior experiments have shown that exposure to crime-related unethical behavior does not increase cheating^40^.

#### Empirical strategy

The following fixed-effect OLS model was used to estimate the causal effect of treatment on self-concept (results shown in Table 2, Column 1),:

1 $$\:{SC}_{t}\:=\:\alpha\:+{\beta\:}_{1}\times\:T+{\beta\:}_{2}\times\:{X}_{t-1}+\:\delta\:+\varepsilon\:$$

In Eq. 1, $\:{SC}_{t}$ is students’ post-treatment self-reported moral self-concept. *T* is the treatment variable, which equals 1 if students answered the treatment questions and 0 if they did not. All control variables are pre-treatment ($\:{X}_{t-1}$) and include gender, age, grade point average (GPA) in the previous semester, and the type, level, and financing of a student’s educational program. The variable $\:\delta\:$ is the study program fixed effect. Standard errors are not clustered, as neither the sampling process nor the treatment assignment process was clustered^41^.

A similar model was used to estimate the effect of the treatment on fraudulent behavior, with results presented in Table 2, Column 2. The key difference between Eq. 1 and Eq. 2 is the dependent variable, which is moral self-concept in Eq. 1 and the sum of the ten die rolls that students reported in Eq. 2.

2 $$\:{Y}_{t}\:=\:\alpha\:+{\beta\:}_{1}\times\:T+{\beta\:}_{2}\times\:{X}_{t-1}+\:\delta\:+\varepsilon\:$$

A two-stage instrumental variable (IV) approach was employed to estimate the causal effect of self-concept on fraudulent behavior (results shown in Table 2, Column 3). The first step is represented by Eq. 1, while the second step is captured by Eq. 3.

In the second step, we used the following model Eq. 3.:

3 $$\:{Y}_{t}\:=\:\alpha\:+{\gamma\:}_{1}\times\:{\widehat{SC}}_{t}+{\gamma\:}_{2}\times\:{X}_{t-1}+\:\delta\:+\varepsilon\:$$

where $\:{Y}_{t}$ is the sum of the ten die rolls that students reported, $\:\widehat{SC}$ is the predicted value from Eq. 1, $\:{X}_{t-1}$ is the vector of students’ pre-treatment characteristics, and $\:\delta\:$ denotes the study program fixed effect. Standard errors are not clustered. The coefficient $\:{\gamma\:}_{1}$ is of particular interest, as it captures the causal effect of self-concept on fraudulent behavior.

## Supplementary Information

Below is the link to the electronic supplementary material.

Supplementary Material 1

## Data Availability

Supplementary materials, data, and all analytical scripts are publicly available on the project page at the Open Science Framework (OSF): https://osf.io/ks4cg/.
