# Supplementary material for: The positive effect of moral self-concept on fraudulent behavior and the need for moral cleansing
Source: Sci Rep. 2025 Aug 29;15:31898. doi: 10.1038/s41598-025-16403-9 (PMC12397343; doi:10.1038/s41598-025-16403-9)
Supplement: Supplementary file 1 — Supplementary Material 1 [file 41598_2025_16403_MOESM1_ESM.docx]

Online Appendix

Version 18. August 2025

for the paper entitled

The positive effect of moral self-concept on fraudulent behavior and the need for moral cleansing

Tamás Keller^1,2^

[keller.tamas@tk.hu](mailto:keller.tamas@tk.hu)

https://orcid.org/0000-0001-6943-0955

Péter Szakál^3^

[szakal.peter@szte.hu](mailto:szakal.peter@szte.hu)

^1^ ELTE Centre for Social Sciences, Computational Social Science Research Group

^2^ ELTE Centre for Economic and Regional Studies, Institute of Economics

^3^University of Szeged

*Corresponding author*

Tamás Keller

[keller.tamas@tk.hu](mailto:keller.tamas@tk.hu)

Table of contents

[*Appendix tables* 3](#_Toc206438530)

[*Appendix A. Control variables* 5](#_Toc206438531)

[*Appendix B. The translated English version of the short questionnaire completed by treated students before self-concept and cheating were measured* 6](#_Toc206438532)

[*Appendix C. The original Hungarian version of the short questionnaire completed by treated students before self-concept and cheating were measured* 7](#_Toc206438533)

[*Appendix D. The translated English version of the die roll task* 8](#_Toc206438534)

[*Appendix E. The original Hungarian version of the die roll task* 11](#_Toc206438535)

## *Appendix tables*

Table A1. Covariate balance: Means for covariates in the control group and the mean difference between the control and treatment groups

|  | Control group  (Mean) | Treated group  (Mean difference) |
| --- | --- | --- |
| Female | 0.70 | 0.03 |
| Age | 23.52 | 0.53 |
| Regular program (=1)  [correspondence program or distance learning (=0)] | 0.81 | 0.03 |
| State-financed (=1)  [self-financed (=0)] | 0.81 | 0.02 |
| Level of degree program  Bachelor’s | 0.62 | –0.05 |
| Master’s | 0.09 | 0.01 |
| Undivided (Master’s equivalent) | 0.19 | 0.05* |
| Advanced vocational training | 0.07 | –0.02 |
| PhD | 0.03 | 0.01 |
| GPA previous semester | 4.19 | 0.07 |
| Admissions score | 363.41 | –3.35 |

**p<0.01, *p<0.05

Table A2. Descriptive statistics for the control variables

|  | Mean | Standard deviation | % missing |
| --- | --- | --- | --- |
| Female | 0.73 | 0.44 | 0 |
| Age | 23.95 | 6.53 | 0 |
| Regular program (=1)  [correspondence program or distance learning (=0)] | 0.84 | 0.37 | 0 |
| State-financed (=1)  [self-financed (=0)] | 0.83 | 0.38 | 0 |
| Level of degree program  Bachelor’s | 0.58 | 0.49 | 0 |
| Master’s | 0.10 | 0.30 | 0 |
| Undivided (Master’s equivalent) | 0.23 | 0.42 | 0 |
| Advanced vocational training | 0.06 | 0.23 | 0 |
| PhD | 0.04 | 0.19 | 0 |
| GPA previous semester | 4.25 | 0.57 | 32.41 |
| Admissions score | 360.7 | 106.6 | 9.17 |

## *Appendix A. Control variables*

The source of the control variables is the university’s register. We attached the control variables to our survey data via students’ ID, allowing us to perform a unique match.^[[1]](#footnote-1)^ We deployed the following baseline variables in all of our estimations:

1. Student’s gender = 1 if the student is female and 0 for if male.
2. Student’s age is the difference between the date of participation and date of birth divided by 365.
3. Student’s GPA is their grade point average calculated from non-missing grades they earned in the previous semester. As first-year students did not have a GPA from the previous semester, we used their admissions score, which is derived from their high school grades. A dummy variable controls for first-year students. A missing GPA is replaced by 0, and a separate dummy variable controls for the missing status.
4. Type of program is a dummy variable indicating whether a student is enrolled in a regular program (=1), on the one hand, or a correspondence program or distance learning (=0), on the other.
5. The student’s education is either state-financed (=1) or self-financed (=0).
6. The level of degree program is coded as Bachelor’s (=1), Master’s (=2), undivided (=3), such as training for physicians and lawyers, and advanced vocational training (4). Advanced vocational training involves two years of training in a higher education institution and results in a qualification similar to a Bachelor’s degree, although the qualification itself is not a degree. We use these variables as separate dummy variables. The Bachelor’s level is the reference category.
7. As fixed effects, separate dummy variables (N=338) indicate the particular study program a student is enrolled in, for example, sociology. Study programs are assigned to university divisions, such as the Faculty of Humanities and Social Sciences.

## *Appendix B. The translated English version of the short questionnaire completed by treated students before self-concept and cheating were measured*

Which person’s behavior do you think better represents your own? On the scale below, 1 means that your behavior is identical to the behavior of person A, and 10 means that your behavior is identical to that of person B. If you use the other numbers on the scale, you will be indicating that your behavior is between that of persons A and B, but closer to one of them.

| **Person A:** S/he tries to get better grades on exams by cheating and other disallowed means. | **Person B:** S/he wouldn’t use such disallowed means even if there were no risk of being caught |
| --- | --- |

| 01 | 02 | 03 | 04 | 05 | 06 | 07 | 08 | 09 | 10 |
| --- | --- | --- | --- | --- | --- | --- | --- | --- | --- |

| **Person A:** When s/he writes an essay for submission, s/he only relies on her own ideas and research. | **Person B:** When s/he writes an essay for submission, s/he incorporates parts of other students’ previously submitted papers without properly indicating it. |
| --- | --- |

| 01 | 02 | 03 | 04 | 05 | 06 | 07 | 08 | 09 | 10 |
| --- | --- | --- | --- | --- | --- | --- | --- | --- | --- |

| **Person A:** His/her motto is: “You don’t have to be honest, just look honest!” | **Person B:** His/her motto is:: “The most important thing is to be honest – I don’t care how others perceive me!” |
| --- | --- |

| 01 | 02 | 03 | 04 | 05 | 06 | 07 | 08 | 09 | 10 |
| --- | --- | --- | --- | --- | --- | --- | --- | --- | --- |

| **Person A:** His/her motto is: “It’s important to follow the rules even if it’s unfavorable to me and hinders my future outcomes.” | **Person B:** His/her motto: “If someone wants to achieve something, s/he’s forced to break certain rules.” |
| --- | --- |

| 01 | 02 | 03 | 04 | 05 | 06 | 07 | 08 | 09 | 10 |
| --- | --- | --- | --- | --- | --- | --- | --- | --- | --- |

## *Appendix C. The original Hungarian version of the short questionnaire completed by treated students before self-concept and cheating were measured*

Az Ön viselkedése az A vagy a B személy viselkedéséhez állna közelebb? Az 1-es azt jelenti, hogy ugyanúgy cselekedne, mint az A jelű személy, a 10-es, hogy ugyanúgy cselekedne, mint a B jelű személy. A többi fokozattal azt tudja érzékeltetni, hogy az Ön cselekvése a kettő között lenne, de valamelyikhez közelebb állna.

| **A. személy:** Puskázással, nem megengedett segédanyagok igénybevételével igyekszik jobb jegyeket szerezni a vizsgákon. | **B. személy:** Akkor sem választ ilyen eszközöket, ha a lebukásnak semmilyen kockázata sincs |
| --- | --- |

| 01 | 02 | 03 | 04 | 05 | 06 | 07 | 08 | 09 | 10 |
| --- | --- | --- | --- | --- | --- | --- | --- | --- | --- |

| **A. személy:** Egy beadandó dolgozat megírásakor csak a saját ötleteire és kutatómunkájára támaszkodik. | **B. személy:** Egy beadandó dolgozat megírásakor jelzés nélkül átemel részeket más hallgatók korábban leadott dolgozataiból. |
| --- | --- |

| 01 | 02 | 03 | 04 | 05 | 06 | 07 | 08 | 09 | 10 |
| --- | --- | --- | --- | --- | --- | --- | --- | --- | --- |

| **A. személy:** Jelmondata: „Nem kell becsületesnek lenned, elég ha annak látszol!” | **B. személy:** Jelmondata: „A legfontosabb, hogy becsületes legyek, az, hogy mások annak látnak-e, nem érdekel!” |
| --- | --- |

| 01 | 02 | 03 | 04 | 05 | 06 | 07 | 08 | 09 | 10 |
| --- | --- | --- | --- | --- | --- | --- | --- | --- | --- |

| **A. személy:** Jelmondata: „A szabályok betartása akkor is fontos, ha ez kedvezőtlen a számomra, és akadályozza a boldogulásomat.” | **B. személy:** Jelmondata: „Aki vinni akarja valamire, rákényszerül arra, hogy egyes szabályokat áthágjon.” |
| --- | --- |

| 01 | 02 | 03 | 04 | 05 | 06 | 07 | 08 | 09 | 10 |
| --- | --- | --- | --- | --- | --- | --- | --- | --- | --- |

## *Appendix D. The translated English version of the die roll task*

Please roll a six-sided die in secret ten times in a row. No one but you will know the values you rolled.

You can use a real die for this task if you have one. Alternatively, you can click any of the following external links, which will open a new page in your browser where you can roll a virtual die [Link1,^[[2]](#footnote-2)^ Link2,^[[3]](#footnote-3)^ Link3^[[4]](#footnote-4)^].^[[5]](#footnote-5)^ You may also open these webpages on your mobile device. In short, you have many options to make sure that nobody knows the number you have rolled.

Each time you roll the die, please report the number you have rolled. The number you report will earn you points. The table below shows you how the number you report corresponds to the points you earn in that round. For example, if you report 1, you earn 0 points in that round. If you report 6, you earn 5 points.

| Number rolled | Point(s) earned |
| --- | --- |
| 1 | 0 |
| 2 | 1 |
| 3 | 2 |
| 4 | 3 |
| 5 | 4 |
| 6 | 5 |

The sum of the points you collect during the ten rounds determines your chance of winning a voucher worth Ft3,000 in the lottery at the end of the questionnaire. We will customize the draws individually for each questionnaire that is started, so there is no maximum number of vouchers that can be won. This means that your chances of winning are not affected by how many people have won before you. Your chances are solely affected by the numbers you report after rolling the die. Winners will be notified by e-mail at the end of the questionnaire. The e-mail will include the online voucher as an attachment.

You can redeem the voucher for online purchases in a wide range of shops, such as sports and leisure, beauty, and fashion shops. You can find the list of online partners in the following table (LINK). The voucher can also be spent on offline purchases in many Hungarian cities. In Szeged, you can buy the voucher offline at the following locations (LINK). For more information on redeeming points, see https://ajikartyak.hu/.

Since you will roll ten times in the game, the game has ten rounds. Your actual chances of winning can be determined at the end of the tenth round. Your chances are equal to the sum of the scores you earn based on the numbers you report during the ten rounds. Your maximum chance of winning is 50%, which you earn if you report rolling 6 in all ten rounds of the game (5×10=50 => 50% chance of winning).

In two examples, the graph below illustrates how your chances of winning vary depending on the value you report as the number rolled in each round:


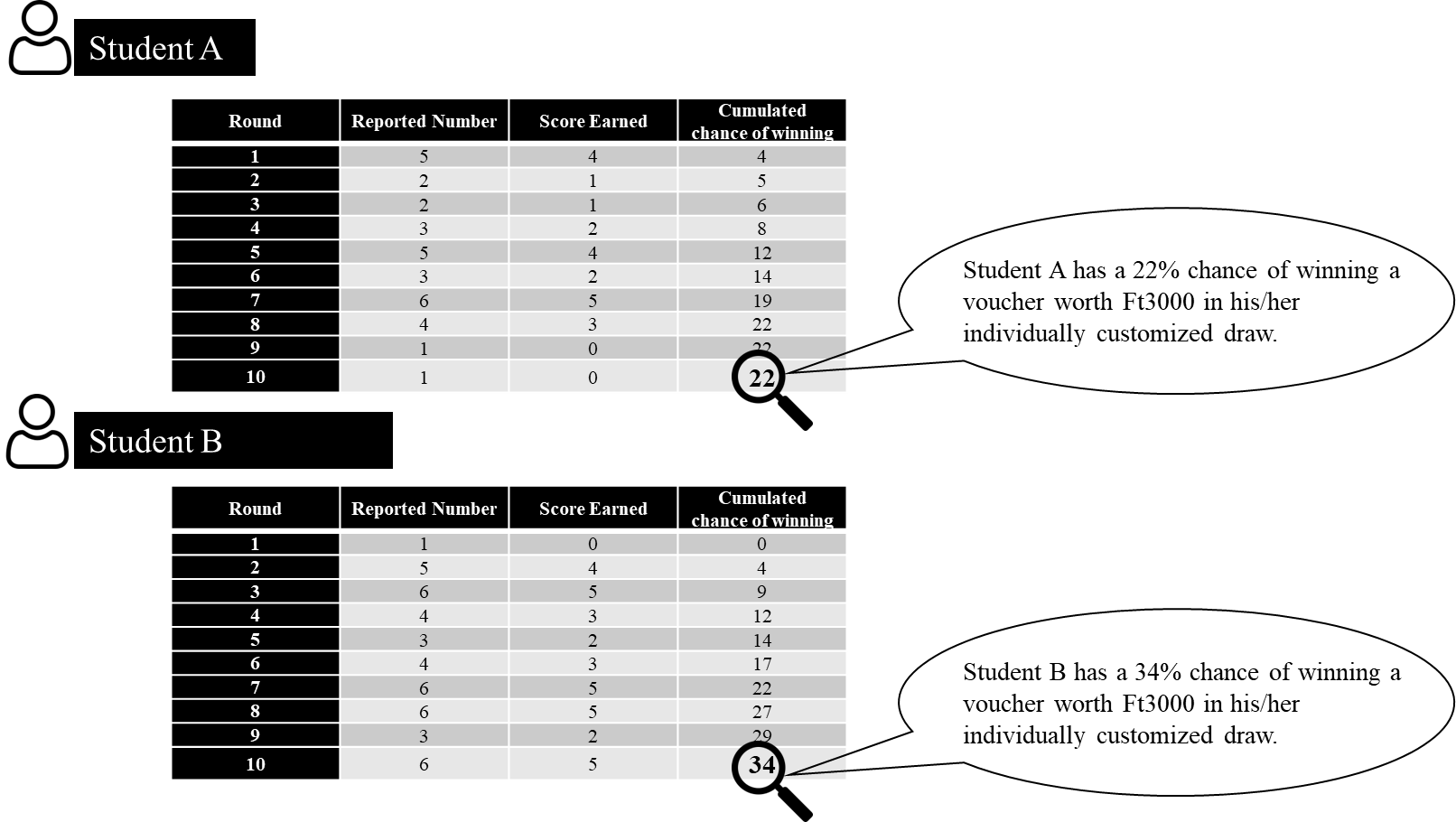


In the first example, Student A reported rolling 5 in Round 1, so s/he scored 4 points in that round, giving him/her a cumulative winning chance of 4% at the end of the round. In Round 2, the same student reported rolling 2, so s/he scored 1 more point in Round 2. In Round 3, the student rolled 2 for a further 1 point, bringing his/her cumulative chance of winning to 4+1+1=5% at the end of that round. Student A’s actual chance of winning can be calculated at the end of the tenth round according to the sum of the points calculated on the basis of the die rolls s/he reported. In this case, Student A has a 22% chance of winning a voucher worth Ft3000 in his/her individually customized draw.

In the second example, Student B reported rolling 1 in Round 1, so s/he earned zero points in that round, with a cumulative chance of winning at the end of that round of 0%. In Round 2, the same student reported rolling a 5, thus earning 4 points in that round, with a cumulative chance of winning at the end of that round of 0+4=4%. At the end of Round 10, Student B’s actual chance of winning is 34%. This is the chance that Student B will win a voucher worth Ft3000 in his/her individually customized draw. This is because s/he collected 34 points in the ten rounds (since 0+4+5+3+2+3+5+5+2+5=34).

In sum, the chance of winning is solely determined by the numbers you enter on the website after each roll of the die. Ultimately, your chance of winning depends on how honest you are when you report the numbers you have rolled.

After we have informed the students of the rules of the game, as described above, they proceed to enter the numbers they have rolled in the table below, which will appear on their screen.

| Round | Number reported | Cumulative chance of winning |
| --- | --- | --- |
| 1 |  |  |
| 2 |  |  |
| 3 |  |  |
| 4 |  |  |
| 5 |  |  |
| 6 |  |  |
| 7 |  |  |
| 8 |  |  |
| 9 |  |  |
| 10 |  |  |

## *Appendix E. The original Hungarian version of the die roll task*

Most arra szeretnénk kérni, hogy dobjon egymás után tízszer egy hat oldalú dobókockával. A dobásai értékét Önön kívül más nem fogja megtudni.

A kockadobásokhoz használhat valódi dobókockát, ha ez rendelkezésére áll. Ezen kívül használhatja az alábbi külső linkeken elérhető virtuális dobókockák egyikét is. A linkekre kattintva böngészőjében egy új ablak nyílik meg. [Link1^[[6]](#footnote-6)^ , Lin2^[[7]](#footnote-7)^ , Link3^[[8]](#footnote-8)^ ] A weboldalakat mobileszközökön is megnyithatja. Összefoglalva, tehát számos lehetőség biztosítja, hogy a kockadobása értékét senki ne tudja meg Önön kívül.

Minden egyes kockadobás után, kérjük, írja be erre az oldalra a dobott számot. Az Ön által beírt számok pontokat érnek. A következő táblázat azt mutatja meg, hogy az Ön által megadott kockadobás értéke alapján Ön hány pontot gyűjtött az adott fordulóban. Például, ha egy adott fordulóban 1-es értéket riportál, akkor abban a fordulóban 0 pontot szerzett. Ha 6-os értéket riportál, akkor 5 pontot kap abban a fordulóban.

| A kockával dobott szám | Megszerzett pontok |
| --- | --- |
| 1 | 0 |
| 2 | 1 |
| 3 | 2 |
| 4 | 3 |
| 5 | 4 |
| 6 | 5 |

A tíz forduló alatt összegyűjtött pontok összege határozza meg azt az esélyt, amellyel Ön a kérdőív végén rendezett sorsoláson 3000 forint értékű utalványt nyerhet. A sorsolást minden megkezdett kérdőív után egyedileg végezzük el. Tehát a megnyerhető vásárlási utalványok száma nincsen maximálva. Vagyis az Ön nyerési esélyeit nem befolyásolja az, hogy Ön előtt korábban hányan nyertek már. Az Ön nyerési esélyeit egyedül az befolyásolja, hogy mekkora számokat írt be a kockadobások értékeként. A nyerteseket a kérdőív végén e-mailben értesítjük. Az e-mail csatolmányként tartalmazni fogja az online vásárlási utalványt.

A vásárlási utalványt számos boltban beválthatja online vásárlások esetében, például sport és szabadidős cikkek, szépség vagy divatárú vásárláskor. Az online partnerek listáját a következő táblázatban találja meg (LINK). Az utalvány offline vásárlások során is elkölthető Magyarország számos városában. Szegeden offline módon a következő helyeken vásárolható le az utalvány (LINK^^[[9]](#footnote-9)^^). További információk a beváltóhelyekről: <https://ajikartyak.hu/>.

Mivel a játék során Ön 10-szer dobhat egymás után, a játéknak 10 fordulója van. Az Ön tényleges nyerési esélye a 10 forduló végére alakul ki, Az Ön nyerési esélye a 10 forduló során beírt kockadobások értéke alapján szerzett pontszámok összegével egyenlő. Az Ön maximális nyerési esélye 50%, ami abban az esetben áll elő, ha Ön mind a tíz fordulóban 6-os dobott értéket adott meg a kockadobás eredményeként (5×10 = 50 => 50% nyerési esély).

A következő ábra két példán keresztül szemlélteti azt, hogy az Ön nyerési esélyei hogyan alakulnak attól függően, hogy milyen kockadobásértéket adott meg az egyes fordulókban:


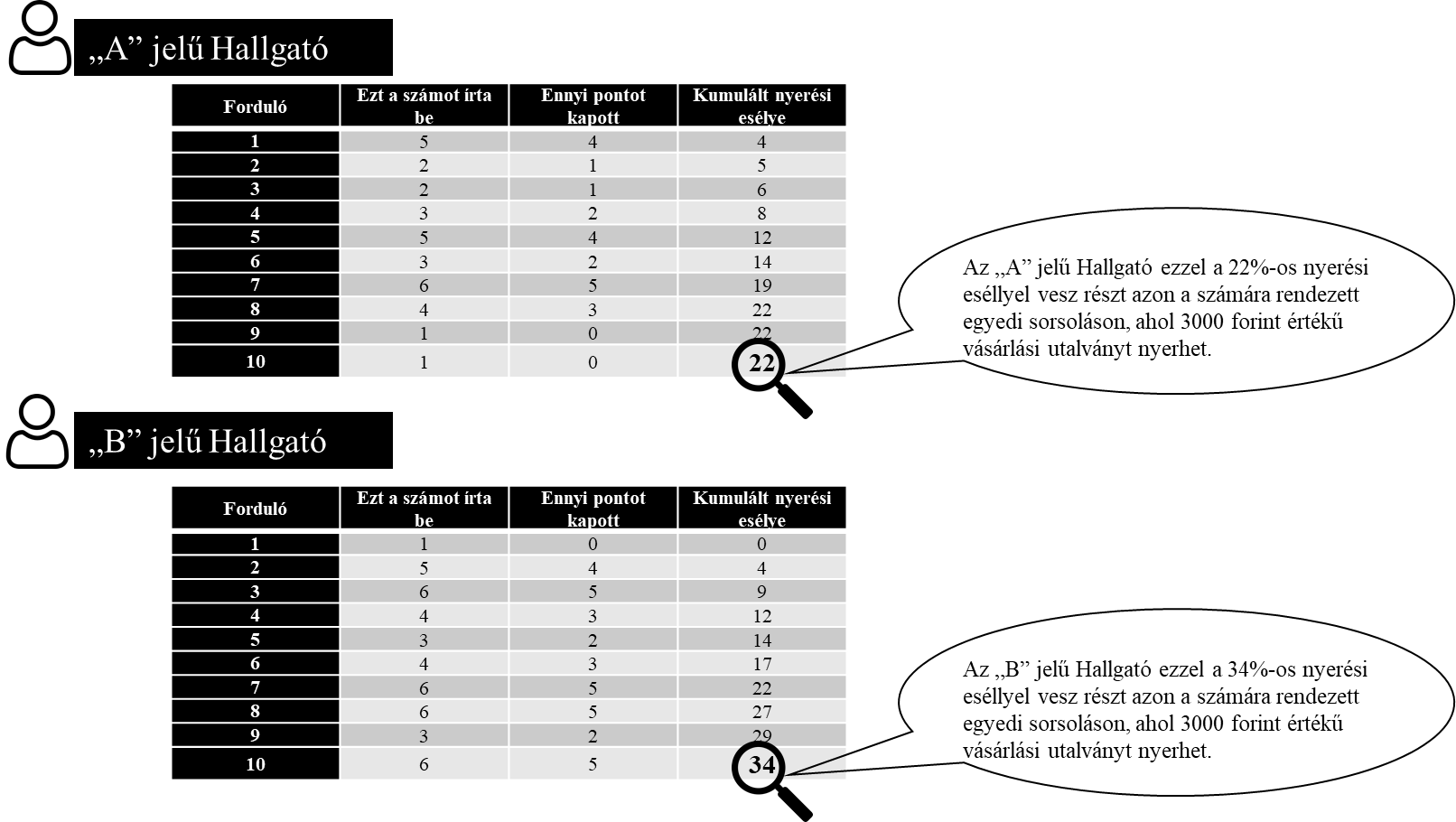


Az első példában szereplő „A” jelű Hallgató az 1. fordulóban 5-ös dobott értéket adott meg, tehát 4 pontot gyűjtött az adott fordulóban, a kumulált nyerési esélye a forduló végén így 4%. A 2. fordulóban ugyanez a Hallgató 2-es dobott értéket írt be, ezzel további 1 pontot szerzett a 2. fordulóban, a kumulált nyerési esélye a forduló végén így 4+1 = 5%. A 3. fordulóban a 2-es dobott érték beírásával még további 1 pontot szerzett a Hallgató, a kumulált nyerési esélye a forduló végén így 4+1+1 = 6%-ra nőtt. Az „A” jelű Hallgató tényleges nyerési esélye a 10. forduló végére alakul ki, mégpedig az általa beírt kockadobások alapján számított pontszámoknak megfelelően. Tehát a példában szereplő „A” jelű Hallgató 22%-os nyerési eséllyel vesz részt azon a számára rendezett egyedi sorsoláson, ahol 3000 forint értékű utalványt nyerhet.

A második példában szereplő „B” jelű Hallgató az 1. fordulóban 1-es dobott értéket adott meg, tehát 0 pontot gyűjtött az adott fordulóban, a kumulált nyerési esélye a forduló végén így 0%. A 2. fordulóban ugyanez a Hallgató 5-ös dobott értéket írt be, ezzel további 4 pontot szerzett az adott fordulóban, a kumulált nyerési esélye a forduló végén így 0+4 = 4%. A 10. forduló végére alakul ki a „B” jelű Hallgató tényleges nyerési esélye, ami 34%. A „B” jelű Hallgató tehát ezzel a nyerési eséllyel vesz részt azon a számára rendezett egyedi sorsoláson, ahol 3000 forint értékű utalványt nyerhet. Mindez azért van így, mert a 10 forduló során 34 pontot gyűjtött, hiszen 0+4+5+3+2+3+5+5+2+5 = 34.

Összefoglalva, az Ön nyerési esélyeit kizárólag azok a számok határozzák meg, amelyeket Ön az egyes kockadobások után a honlapra beírt. Végső soron tehát nyerési esélye attól függ, hogy mennyire becsületes akkor, amikor megadja a kockadobások értékét.

Miután a fent leírtak szerint tájékoztattuk a diákokat a játék szabályairól, a diákok beírják a dobott számokat az alábbi táblázatba, amely megjelenik a képernyőjükön.

| Forduló | Dobott szám | Kumulált nyerési esély |
| --- | --- | --- |
| 1 |  |  |
| 2 |  |  |
| 3 |  |  |
| 4 |  |  |
| 5 |  |  |
| 6 |  |  |
| 7 |  |  |
| 8 |  |  |
| 9 |  |  |
| 10 |  |  |

1. We ensured the integrity of students’ personalized data with a two-stage process of registering for the experiment. In the first step, students received a recruitment e-mail and registered for the experiment. In the second step, students who registered received a personalized weblink, which identified them with unique tokens instead of their sensitive university ID. After background data from the university register was attached to the experiment with a key that matched the tokens and university ID, this key was erased, thus making it impossible to identify students in the university register for a second time. [↑](#footnote-ref-1)
2. <http://kocka.bagoj.hu/> [↑](#footnote-ref-2)
3. <https://rolladie.net/#!start=false> [↑](#footnote-ref-3)
4. <https://www.online-stopwatch.com/chance-games/roll-a-dice/full-screen/> [↑](#footnote-ref-4)
5. The Java scripts for these pages indicate that they are using the same method to generate random numbers. [↑](#footnote-ref-5)
6. <http://kocka.bagoj.hu/> [↑](#footnote-ref-6)
7. <https://rolladie.net/#!start=false> [↑](#footnote-ref-7)
8. <https://www.online-stopwatch.com/chance-games/roll-a-dice/full-screen/> [↑](#footnote-ref-8)
9. https://ajikartyak.hu/elfogadohely?350 (elektrofitness)

   https://ajikartyak.hu/elfogadohely?269 (Ibusz jegyiroda)

   https://ajikartyak.hu/elfogadohely?379 (hőlégballon)

   https://ajikartyak.hu/elfogadohely?107 (Dockyard) [↑](#footnote-ref-9)
